# Predictors of and reasons for pacifier use in first-time mothers: an observational study

**DOI:** 10.1186/1471-2431-12-7

**Published:** 2012-01-19

**Authors:** Chelsea E Mauch, Jane A Scott, Anthea M Magarey, Lynne A Daniels

**Affiliations:** 1 Nutrition and Dietetics, School of Medicine, Flinders University, Adelaide, South Australia, Australia; 2 Institute of Health and Biomedical Innovation, Queensland University of Technology, Brisbane, Queensland, Australia

## Abstract

**Background:**

The use of pacifiers is commonplace in Australia and has been shown to be negatively associated with breastfeeding duration. In order to influence behaviour related to the use of pacifiers it is important to understand the reasons for their use. The primary aim of this observational study was to investigate who (if anyone) advises first-time mothers to give a pacifier and the reasons for which they first give (or try to give) a pacifier to their infant. Additionally, this study investigated the predictors of pacifier use and the relationship between pacifier use and breastfeeding duration.

**Methods:**

In total, 670 Australian first-time mothers recruited as part of the NOURISH trial completed a questionnaire regarding infant feeding and pacifier use.

**Results:**

Pacifiers were introduced by 79% of mothers, of whom 28.7% were advised to use a pacifier by their mother/mother-in-law with a further 22.7% being advised by a midwife. The majority of mothers used a pacifier in order to soothe their infant (78.3%), to help put them to sleep (57.4%) and to keep them comforted and quiet (40.4%). Pacifiers given to infants before four weeks (adjHR 3.67; 95%CI 2.14-6.28) and used most days (adjHR 3.28; 95%CI 1.92-5.61) were significantly associated with shorter duration of breastfeeding.

**Conclusions:**

This study identifies an opportunity for educating new mothers and their support network, particularly their infant's grandmothers, with regards to potential risks associated with the early and frequent use of a pacifier, and alternative methods for soothing their infant, in order to reduce the use of pacifiers and their potentially negative effect on breastfeeding duration.

## Background

Breastfeeding is known to be the ideal form of infant nutrition, not only because of its direct nutritional benefits to the infant, but also for its immune-protective and numerous other physiological benefits to the infant and mother [1,2]. In Australia, results of the 2004-2005 National Health Survey indicate that while 87.8% of mothers initiated breastfeeding, only half of infants (50.4%) were being breastfed to some extent at 6 months of age [3]. Pacifier use has been shown to have a strong negative association with decreased exclusive and overall breastfeeding duration [4,5]. The early introduction of a pacifier rather than pacifier use *per se *appears to be strongly associated with shortened duration of breastfeeding. One of few randomized controlled trials (RCT) investigating this association reported a shorter overall breastfeeding duration in infants introduced to the pacifier by four weeks compared to those introduced from five weeks (adjHR 1.22; 95% CI 1.03 - 1.44) [6]. Similarly, a longitudinal study from Australia found shorter duration of breastfeeding to be associated with pacifier introduction prior to but not after 10 weeks of age [7]. A dose-related effect has been observed in four observational studies, where frequent pacifier use shows a stronger negative association with breastfeeding duration than occasional or infrequent use [8-11].

Despite this observational evidence, a recent systematic review reported that four RCTs with interventions aimed at reducing pacifier use did not demonstrate a difference in breastfeeding outcome [12]. The interventions included 'no pacifier' use and education regarding the avoidance of pacifiers and alternative soothing methods compared with education regarding soothing methods including pacifier use [9,13-15]. However, in two of these studies, non-compliance with the intervention was high, with many mothers in the intervention groups choosing to use a pacifier [12], and thus the interventions were not delivered as intended.

Little is currently known about the reasons behind the use of pacifiers, and whether or not they have simply become a cultural norm [7,8,11]. The poor compliance in intervention studies suggests that the use of pacifiers is firmly entrenched in some cultures and that the reasons why mothers use pacifiers needs to be investigated and better understood in order to design effective interventions to reduce pacifier use. Hence the aim of this study was to investigate who (if anyone) advises first-time mothers to give a pacifier and the reasons for why they first give (or try to give) a pacifier to their healthy term infant. Additionally, this study aimed to identify predictors of pacifier use and confirm (or refute) previous research regarding pacifier use and breastfeeding duration.

## Methods

### Sample

**Figure 1:**
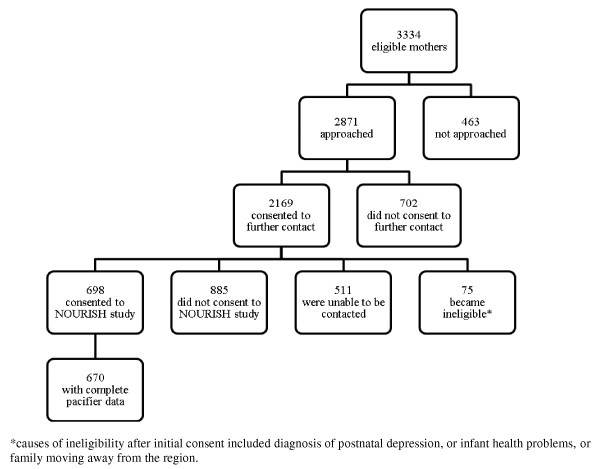
**Subject Recruitment Flowchart**. Figure 1 *causes of ineligibility after initial consent included diagnosis of postnatal depression, or infant health problems, or family moving away from the region.

Participants were mothers and infants enrolled in the NOURISH study which has been described elsewhere [16]. NOURISH is a multi-centre, RCT evaluating the efficacy of a community-based intervention that encouraged positive feeding practices that promote healthy infant food preferences and intakes (Australasian Clinical Trials Registration ACTRN 1260800056392)[16]. Subjects were recruited in a two-phase process in Adelaide, South Australia and Brisbane, Queensland, Australia (Figure 1). A consecutive sample of eligible mothers was first approached in public and private hospitals, after delivery of their infant from February until June 2008 and September 2008 until March 2009. The first approach requested consent and details for later contact. Consenting mothers were contacted again when their baby reached four to seven months for full enrolment and baseline assessment prior to allocation to the trial. Eligibility criteria included medically healthy primiparous birth, infant born at 37 or more weeks gestation and birth weight of at least 2500 g; mother aged 18 years or more with good written and verbal English skills and residing in (or near) Adelaide or Brisbane. Mother-infant pairs were excluded if the infant was diagnosed with a congenital abnormality or chronic condition that was likely to influence development, including feeding ability, or if the mother self-reported eating or mental health disorders. Approval was obtained from the ethics committees at both Flinders University and Queensland University of Technology, and each recruitment hospital.

### Data collection

Data collected at first contact from both consenting and, where possible, non-consenting mothers included maternal age and relationship status at infant's birth, highest level of education attained, country of birth and infant gender. Those consenting to full enrolment completed the baseline assessment which included a self-administered questionnaire. A combination of evidence from the literature and expert opinion was used to develop four questions on pacifier use. One open-ended question asked for the age at which the pacifier was first given. Three pre-coded questions considered frequency of pacifier use, who (if anyone) advised the mother to give her infant a pacifier and the reasons for giving a pacifier. An '*other' *category was included in the pre-coded questions to ensure that all responses were captured.

### Data analysis

All data were double-entered into a Microsoft Access database, and then imported into the Statistical Package for the Social Sciences (SPSS 17.0). Study participants were compared with non-participants using independent samples T-Tests for maternal age at infant's birth, and Chi-square tests for independence for relationship status at infant's birth, highest level of education attained, country of birth and initial feeding intention. Non-participants were those mothers who declined further contact at the first approach but consented to provide basic demographic data, and those who subsequently declined, or could not be recontacted for, full enrolment. Descriptive statistics were used to present data on variables related to pacifier use.

Bivariate and multivariate binary logistic regression analyses were used to investigate pacifier use in relation to mother's age and relationship status at infant's birth, highest level of education, country of birth, and infant's gender. Infants were defined as pacifier 'users' or 'non-users' (dependent variable) based on whether or not the infant had ever used a pacifier, regardless of current use. Bivariate Cox regression analyses were used to determine hazard ratios (HR) and 95% confidence intervals (CI) for breastfeeding duration by age pacifier given and extent of pacifier use, maternal age, level of education, relationship status and infant gender. Breastfeeding duration refers to the duration of time that a mother breastfed to any extent and was measured in weeks. To account for those still breastfeeding at baseline, a censoring factor was applied. Two multivariate Cox regression analyses were performed to determine if age pacifier given (model one) and the extent of pacifier use (model two) were independently associated with breastfeeding duration adjusting for potential confounding factors demonstrating a significant association with breastfeeding duration in the bivariate analyses. Survival analyses (Life Tables) plots were used to illustrate the effect of age at pacifier introduction and the extent of pacifier use on breastfeeding duration. Pairwise comparisons were made in order to determine if there were significant differences between survival curves. Significance for all analyses was set at a P-value of 0.05 or less.

For both logistic and Cox regression analyses, maternal age was categorised into four age groups; < 25 years, 25 to 29 years, 30 to 34 years and 35+ years (reference). Relationship status was collapsed into two categories; 'in a relationship' (reference) and 'not in a relationship'. Highest level of maternal education was collapsed into three categories; university (reference), Trade / Technical And Further Education (TAFE), and high school. Mother's country of birth was grouped into 'Australia' (reference) and 'Other'.

## Results

### Sample characteristics

A total of 670 mothers from the full allocate NOURISH sample (N = 698), of whom 63% were from Brisbane, provided complete data required for this study. One third of participants were aged between 30 and 34 years (n = 243) and 78 per cent were born in Australia (n = 522) (Table 1). The mean age of the infants at baseline was 18.6 weeks (± 4.3, range 9.4 - 31.6) with 97% being breastfed at some stage since birth and 73.2% still breastfed to some extent at the time the questionnaire was completed (57.8% fully breastfed, 15.4% breast plus formula). When compared to non-participants, participants were significantly older (30.8 ± 5.2 vs 27.9 ± 5.5 years, P < 0.001), had a higher level of education (χ^2 ^168.00, P < 0.001), were more likely to be in a relationship (χ^2 ^29.31, P < 0.001) and more likely to intend to breastfeed (χ^2 ^19.31, P < 0.001).

**Table 1:** Characteristics of participants and non-participants

Variable	Participants (N = 670*)	Non-participants (N = 1780*)	Pearson Chi Square Value
	
	Mean (SD) or N (%)		(P Value)
**Mother's age at delivery**	30.8 (5.2)	27.9 (5.5)	(< 0.001)
**Highest level of education**			
University	395 (59.0)	545 (31.8)	168.00
Trade / TAFE^a^	154 (23.0)	457 (26.7)	(< 0.001)
High School	121 (18.0)	710 (41.5)	
**Mother's relationship status at infant's birth**			
Not in a relationship	30 (4.5)	200 (11.8)	29.31
In a relationship	639 (95.5)	1493 (88.2)	(< 0.001)
**Initial feeding intention**			
Breastfed	628 (94.3)	1565 (88.8)	19.31
Formula	10 (1.5)	85 (4.8)	(< 0.001)
Combination (breastfed & formula)	28 (4.2)	113 (6.4)	
**Mother's country of birth**			
Australia	522 (78.6)	1288 (75.8)	2.10
Other	142 (21.4)	411 (24.2)	(0.16)

^a ^Technical And Further Education (TAFE)

*totals may not add up to N value due to missing data in some variables

### Pacifier use

In total 79% of infants (n = 532) had ever used a pacifier, and 69% of infants (n = 464) were currently using a pacifier at baseline, while 10% of mothers had tried but were no longer giving a pacifier to their infant. The median age at which a pacifier was introduced was 2 weeks (IQR 0.6-4 weeks) and two thirds of infants (n = 353) were given a pacifier prior to 4 weeks of age. Of those infants currently using a pacifier, 85.1% (n = 395) were using it most days and 14.9% less often. The frequency of pacifier use was not associated with the age at which it was first given (χ^2 ^3.43, P = 0.18). Mothers with a high school education, compared with a university education, were more likely to give their infant a pacifier (OR 2.12; 95% CI 1.17 - 3.81) and mothers born outside Australia were less likely to use pacifiers (OR 0.60; 95% CI 0.39 - 0.93) (Table 2). The relationship with mother's highest education level and country of birth remained significant (adjOR 1.95; 95% CI 1.08 - 3.53 and adjOR 0.61, 95% CI 0.40 - 0.95, respectively) after adjustment for potential confounders.

**Table 2:** Bivariate and multivariate logistic regression: factors associated with pacifier use (N = 670)

Variable	Crude Odds Ratio (95% Confidence Interval)	Adjusted Odds Ratio (95% Confidence Interval)
**Mother's age at delivery**		
< 25 years	1.03 (0.54 - 2.00)	
25-29 years	1.11 (0.65 - 1.91)	
30-34 years	0.91 (0.55 - 1.50)	
≥35 years	1.00	
**Highest level of education**		
University	1.00	1.00
Trade / TAFE	1.14 (0.73 - 1.80)	1.12 (0.70 - 1.77)
High School	2.12 (1.17 - 3.81)	1.95 (1.08 - 3.53)
**Mother's relationship status at infant's birth**		
Relationship	1.00	
Not in a relationship	1.31 (0.49 - 3.50)	
**Infant's gender**		
Female	1.00	
Male	1.34 (0.92 - 1.95)	
**Mother's country of birth**		
Australia	1.00	1.00
Other	0.60 (0.39 - 0.93)	0.61 (0.40 - 0.95)

### Who advised mother to give a pacifier and the reasons for first giving (or trying to give) a pacifier?

Approximately one third of mothers (30.6%) reported that no-one had advised them to use a pacifier, while mothers or mothers-in-law, and midwives were the most common sources of advice (28.7% and 22.7% respectively) (Table 3). Friends were an important source of advice (20.2%) with other family members (16.6%) and husbands/partners (14.7%) less so. A small number of women were advised by a medical professional or other health professional to use a pacifier. Mothers generally reported more than one reason for giving their infant a pacifier (Table 4). The most common reasons were to soothe their infant (78.3%), to help put them to sleep (57.4%) and to keep them comforted and quiet (40.4%). One in five mothers introduced a pacifier 'because it is natural for babies to suck' and a further one in five introduced it to prevent their baby from sucking their thumb. A number of reasons related to breastfeeding were also selected, namely to stretch the length of time between feeds, to help take baby off the breast after a feed, and to reduce non-nutritive sucking time on the breast. It was also used to soothe babies when teething.

**Table 3:** Who advised the mother to give her infant a pacifier (multiple response frequencies) (N = 529)

Advised mother to give infant a pacifier	Percentage of cases (%)
**Prompted responses**	
No-one	30.6
Mother / mother-in-law	28.7
Midwife	22.7
Friend(s)	20.2
Other family member	16.6
Husband / partner	14.7
Child health nurse	9.8
Doctor / GP	3.2
**Unprompted (other) responses**	
Other health professional	2.6
Other / Unspecified person	1.2
Given to infant by hospital staff without permission	0.4

**Table 4:** Reasons for first giving (or trying to give) infant a pacifier (N = 530)

Reasons for first giving (or trying to give) infant a pacifier	Percentage of cases*
**Prompted responses**	
To soothe baby when upset/irritable, or for other reasons	78.3
To help put baby to sleep	57.4
To keep baby comforted and quiet	40.4
Because it is natural for babies to suck	21.9
To prevent baby from sucking thumb	20.9
To help stretch the time between feeds	12.6
To soothe baby when teething	9.4
To help in taking baby off the breast after a feed	6.8
As a distraction	6.2
Because it reduces baby's risk of SIDS	4.7
Because it is normal to use a pacifier	1.9
To help wean baby from breast to bottle	0.9
Don't know the reason	0.4

**Unprompted (other ) responses**	
To treat/reduce baby's reflux/vomiting/colic/wind/hiccups	4.3
To reduce 'non-nutritive' sucking on breast	3.4
To assist in / improve attachment / breastfeeding	1.7
Other reasons/not specified	1.4
To reduce the effect of pressure changes during flights	0.8

* Mothers could cite more than one reason

### Pacifier use and breastfeeding duration

**Figure 2:**
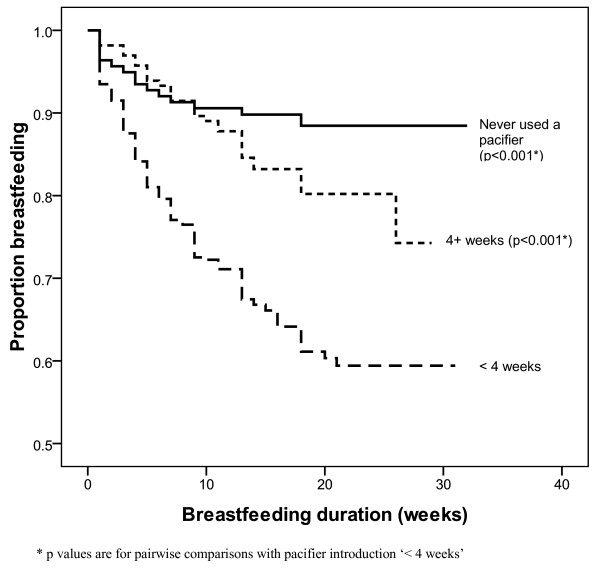
**Survival curve - breastfeeding duration by age at pacifier introduction**. Figure 2 footnote * p values are for pairwise comparisons with pacifier introduction '< 4 weeks'.

**Figure 3:**
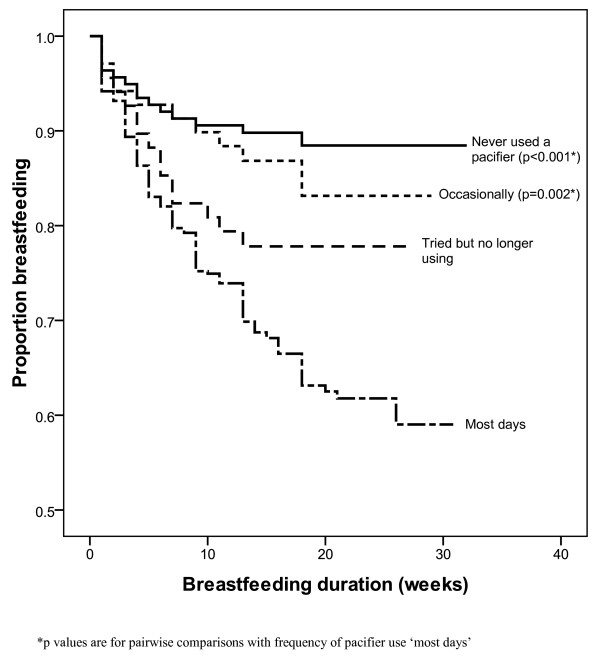
**Survival curve - breastfeeding duration by extent of pacifier use**. Figure 3 footnote *p values are for pairwise comparisons with frequency of pacifier use 'most days'.

After adjusting for mother's highest level of education, mother's age at delivery and relationship status in the Cox regression analyses, mothers who gave (or tried to give) their infant a pacifier prior to 4 weeks of age were more likely to have discontinued breastfeeding (adjHR 3.67; 95% CI 2.14 - 6.28) than mothers who had never given their infant a pacifier. Similarly, in a second adjusted model use of a pacifier on most days was significantly associated with shorter duration of breastfeeding (adjHR 3.28; 95% CI 1.92 - 5.61) compared with never having used a pacifier. Survival curves for overall breastfeeding duration by age of introduction of pacifier and extent of pacifier use are displayed in Figures 2 and 3.

## Discussion

There is limited published research regarding who and what influences a mother's decision to give her infant a pacifier. In view of the well documented, and confirmed in this study, negative association between pacifier use and breastfeeding duration, the results of this study are of importance to inform the design of any future interventions aimed at reducing pacifier use.

This study confirms the widespread use of pacifiers by Australian mothers reported in an earlier study [17] with eight out of 10 infants having been given a pacifier at some stage and seven out of 10 infants still using a pacifier at the time data for this study were collected. In the present study, women with a high school education were more likely to use a pacifier than women with a university education. This association between lower education level and pacifier use is supported by a previous study conducted in Brazil [18]. Cultural differences in the use of pacifiers were observed in this current study with women born outside of Australian being significantly less likely to give their infant a pacifier than Australian born mothers. This finding is consistent with the findings of a multicentre study [19] which reported a widespread difference between countries in the prevalence of pacifier use ranging from 12.5% in Japan to 71% in the Ukraine. The findings of this study suggest that pacifier use in Australia, while approaching universality, is still significantly influenced by socioeconomic and cultural factors.

This study showed that almost 60% of mothers gave (or tried to give) their infant a pacifier to help settle them to sleep, which is consistent with the findings of a New Zealand study [11]. Using pacifiers to soothe or comfort a crying or distressed infant, or to settle an infant to sleep, is likely to result in prolonged or extensive use of the pacifier as crying and sleep are both necessary and frequent behaviours in newborn infants. This may explain the early introduction of pacifiers by the majority of mothers (two thirds before 4 weeks) and the large percentage of mothers using pacifiers most days. The reportedly wide variation in the use of a pacifier between different countries [19] suggests that women from other cultures must use other methods that do not involve the use of a pacifier to effectively soothe their infant. Certainly, it has been shown that pacifiers are no more effective than the traditional ('attachment') methods of soothing (breastfeeding, carrying, rocking) [9] and the traditional methods of soothing may better support mother-infant bonding and subsequently breastfeeding success [20].

Another reason why relatively large numbers (20%) of mothers introduced a pacifier was 'because it is natural for babies to suck'. A similar reason was cited in a New Zealand study with almost half of the mothers reporting they used the pacifier to satisfy their infant's 'need' to suck [11]. One of five mothers also gave their infant a pacifier to prevent them from sucking their thumb. However the use of a pacifier to discourage thumb sucking may replace one bad habit with another, both of which have been shown to increase the risk of dental malocclusion [21]. A recent study reported that for each additional year of persistence with non-nutritive sucking, either pacifier use or finger sucking, there was a 2.3 times greater chance of dental malocclusion [22].

Finally, some mothers intentionally used the pacifier in order to stretch the time between breastfeeds (13%), to help remove the baby from the breast after a feed (6.8%) or to reduce 'non-nutritive' (or 'comfort') sucking on the breast (3.4%). It is important that breastfed infants are demand fed in the first weeks of life in order to establish the breast milk supply [23]. The use of a pacifier for these reasons, particularly in the first four weeks of life, may disrupt the establishment of milk supply, thereby leading to a shorter duration of breastfeeding. The use of a pacifier to prolong the time between breastfeeds may reflect a mother's desire or naïve expectation of autonomy from their infant. This is consistent with the finding in numerous studies that women who choose to partially breastfeed do so in order that they can leave their child in the care of their partner or another person [24] or discontinue breastfeeding due to a sense of restriction [25].

The use of pacifiers may be related to a variety of inter-related factors. For instance, younger, less educated mothers may be less aware of alternative methods of soothing infants, whereas older, better educated mothers may use 'attachment methods' of soothing such as carrying, rocking, swaddling, singing and massage and only use 'non-attachment methods' of soothing (i.e. pacifiers) as a last resort. A Dutch multicultural study demonstrated that less educated women were less likely to carry, rock or swaddle their infant and more likely to give their infant a pacifier or night bottle compared with more educated women [26]. It may be that the concomitant use of alternative non-attachment soothing methods such as a night bottle may be a confounder not considered in this or other studies. While plausible mechanisms for how a pacifier may contribute to the early cessation of breastfeeding have been postulated it is possible that the use of a pacifier may simply be a marker of breastfeeding problems that result in the early cessation of breastfeeding rather than an independent cause of breastfeeding cessation [10]. For instance, prolonged suckling at the breast may be an indicator that an infant's nutritional needs are not being met, perhaps due to poor feeding technique, and warrants investigation by a health care professional.

Studies have shown that an infant's grandmothers (both maternal and paternal) are a key influence on the way a first-time mother cares for her child and they have been shown to be influential with regards to a woman's decision to initiate [27] and continue breastfeeding [7]. They may be a source of both solicited and unsolicited advice, and in this study mothers and mothers-in-law were identified as a woman's primary source of advice regarding the use of a pacifier. Many of these grandmothers may not be aware of the negative association of pacifier use with breastfeeding duration and dental malocclusion, as much of this evidence has been published in the last 20 years or so. Interventions that aim to reduce the use of pacifiers should include opportunities for grandmothers to learn of the risks associated with early and frequent pacifier use. These may be in the form of print material specifically targeted at grandmothers and/or the opportunity to accompany their daughters/in-law to antenatal classes where feeding and pacifier use is discussed, to ensure that their knowledge aligns with current recommendations.

A third of mothers reported being advised to use a pacifier by a midwife or child health nurse. The questionnaire design did not allow for identification of whether women had received this advice from a hospital-based or community-based midwife or child health nurse. Based on the 'Ten steps to successful breastfeeding', Baby Friendly Health Initiative (BFHI) accredited hospitals discourage the use of artificial teats or pacifiers in breastfeeding mothers [23]. Future research should distinguish between hospital-based and community-based workers in order to investigate further the association between the advice and BFHI accreditation. Nevertheless, ensuring the currency and quality of midwife and child health nurse advice is important.

This study confirmed the findings of numerous other studies that there is a negative association between pacifier use and breastfeeding duration, and more specifically, that the association is related to the time of introduction and frequency of use. Our results indicate that infants given a pacifier prior to four weeks of age and those using pacifiers most days had a three-fold risk of shorter breastfeeding duration, independent of maternal education and age. These results are similar to those of two Australian studies, supporting a stronger association between shorter breastfeeding duration and early pacifier introduction compared with later introduction [7,17]. Previous research has also found similar results with regards to frequency of use [8,10].

This study has a number of limitations, firstly the restriction of this study to first time mothers means that results cannot be generalised to all mothers, although previous research indicates that the association between pacifier use and breastfeeding duration exists in both primiparous and multiparous mothers [5]. A major limitation of the study is that the sample is not representative of the population from which it was drawn, further limiting the generalizability of results. While 76% of women contacted shortly after delivery agreed to be contacted when their infants were older, only 44% consented to participate further when approached the second time. The relatively low response rate is consistent with other Australian studies that involve an active intervention [28,29]. First time mothers were probably less inclined, once they had realised how time consuming caring for a young infant can be, to participate in a study which possibly would require them to attend education sessions. However this also means that the significance of some results to the general population may have been underestimated. For example, participants were older and better educated and likely therefore to be more health conscious. Given that an education level lower than university was positively associated with both early introduction and more frequent use of pacifiers, this may lead to an underestimation of both these measures of pacifier use. The investigation of pacifier use was not the primary purpose of the NOURISH study, which limited the ability to investigate pacifier use more extensively, particularly because the design of the study was retrospective, introducing potential recall bias, and preventing investigation of the causality of the relationship between pacifier use and breastfeeding duration.

Nevertheless, this study has several strengths and confirms the findings of earlier studies. As previously identified breastfeeding rates and rates of pacifier use vary greatly between countries [19], highlighting the need for country specific data, which this study provides being one of only a handful of studies conducted in Australia. The sample size of this study was relatively large, and inclusion of data from two cities increases its generalizability. The scope of this study is greater than previous Australian studies, being the first to investigate both who advises first-time mothers to give a pacifier, and the reasons for which they first give a pacifier to their infant.

## Conclusions

This study confirms the findings of earlier studies that the use of a pacifier is widespread in Australia and that the early introduction, and frequent use, of a pacifier is associated with shorter breastfeeding duration. Furthermore, it identifies an opportunity for educating new mothers and their support network, particularly grandmothers, with regards to potential risks associated with the early and frequent use of a pacifier, and alternative methods for soothing their infant, in order to reduce the use of pacifiers and the potentially negative effect associated with their use on breastfeeding duration.

## Competing interests

The authors declare that they have no competing interests.

## Authors' contributions

LD took the leading role in designing the NOURISH study and writing the grant that was subsequently funded by the National Health and Medical Research Council and commented on drafts of this manuscript. AM contributed to the study design and grant preparation and commented on drafts of this manuscript. CM developed the pacifier related questions, conducted the analysis for this study and wrote the first draft of the manuscript. JS contributed to the development of the pacifier related questions, supervised the analysis for this study, and edited the first and subsequent drafts of the manuscript. All authors read and approved the final manuscript.

## Pre-publication history

The pre-publication history for this paper can be accessed here:

http://www.biomedcentral.com/1471-2431/12/7/prepub
